# Supplementary material for: A deep learning-based approach to enhance accuracy and feasibility of long-term high-resolution manometry examinations
Source: Commun Med (Lond). 2025 Dec 2;5:513. doi: 10.1038/s43856-025-01255-1 (PMC12678422; doi:10.1038/s43856-025-01255-1)
Supplement: Supplementary file 2 — Supplementary Material [file 43856_2025_1255_MOESM2_ESM.pdf]

A deep learning based approach to enhance accuracy and feasibility of long-term high-resolution manometry examinations

Supplementary information

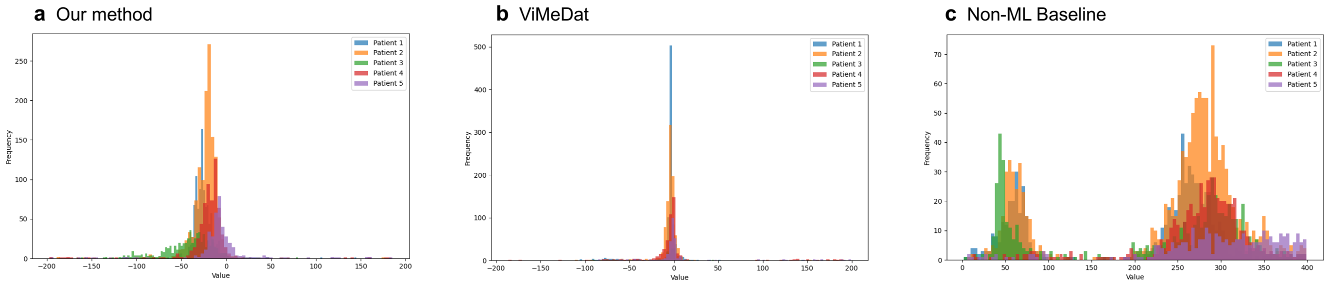

Supplementary Figure 1. The distance between the detected swallow and the correct swallow start for 5 patients. Distances of outputs of our MobileNet based approach are centered around -35, indicating that this approach is typically predicting the start of a swallow slightly earlier compared to the labelled start. The distances of the ViMeDat outputs are centered around 0, indicating that the software is typically predicting the swallow exactly at the true swallow start. The baseline, as it is designed to identify high pressure events which mostly occur during the swallow, is typically predicting the swallow event after the swallow start. It can be observed that most of the times a high pressure event is either occurring rather soon after the labeled swallow start (50 measurements or 1 second), or slightly (around 270 measurements or 5-6 seconds) after the swallow start.
