# Supplementary material for: A deep learning-based approach to enhance accuracy and feasibility of long-term high-resolution manometry examinations
Source: Commun Med (Lond). 2025 Dec 2;5:513. doi: 10.1038/s43856-025-01255-1 (PMC12678422; doi:10.1038/s43856-025-01255-1)
Supplement: Supplementary file 3 — Description of additional supplementary file [file 43856_2025_1255_MOESM3_ESM.pdf]

Description of additional supplementary file

File name: Supplementary Data

Description: Source data underlying Figure 8, Table 1, Table 2 and Table 3
